# A Novel Method for the Accurate Measurement of Soil Infiltration Line by Portable Vector Network Analyzer

**DOI:** 10.3390/s21217201

**Published:** 2021-10-29

**Authors:** Xiaobin Li, Zhengguang Liu, Lei Lin, Hao Fan, Xingyu Liang, Jinghui Xu

**Affiliations:** Key Laboratory of Agricultural Soil and Water Engineering in Arid and Semi-Arid Areas, Ministry of Education, Northwest A&F University, Xianyang 712100, China; Li1996@nwafu.edu.cn (X.L.); zhengguangliu@nwafu.edu.cn (Z.L.); 2019050865@nwafu.edu.cn (L.L.); fh0213@nwafu.edu.cn (H.F.); lxy173037841@nwafu.edu.cn (X.L.)

**Keywords:** VFTT, soil infiltration line, portable vector network analyzer, dielectric measurement, time domain to frequency domain conversion, impedance change

## Abstract

Accurate measurement of soil infiltration lines is very important for agricultural irrigation systems. It can help monitor the irrigation of soil to control irrigation amounts and promote crop growth. The soil infiltration line is a complex dynamic boundary and is difficult to model accurately, leading to estimation deviation. A traditional TDR (time domain reflectometry) method is used in soil infiltration line measurement, but it lacks good applicability and accuracy. In this paper, we proposed a method—VFTT (The vector network analyzer’s frequency domain signals are converted to the time domain)—by the time domain to frequency domain conversion principle to improve the accuracy of soil infiltration line measurement. The experiment results show that the measurement method of soil infiltration line based on VFTT has high accuracy and robustness. After fitting the measured value with the actual one, R^2^ reaching more than 0.98 can effectively measure the position of the soil infiltration line.

## 1. Introduction

During soil irrigation, the soil infiltration line is a visible boundary between the surface of seepage flow and dry soil [1]. However, the precise positioning of this line is still a big challenge. Accurate monitoring of the position of the infiltration line of farms can help discover the law of soil water movement and improve the effect of water-saving irrigation [2,3,4]. It can improve the level of automation in irrigation decision-making [5,6,7,8,9,10]. It is useful in preserving agricultural ecology [11,12,13], raising water-saving irrigation levels [14,15], and implementing intelligent irrigation [16,17].

In recent years, the study of soil infiltration lines mainly focused on the interaction between crops and soil [18,19]. There are still some deficiencies in determining the position of the wetted line [20,21]. Shiri et al. [22] observed water infiltration in the drip irrigation system in the transparent test device and used the random forest method (RF) to simulate the position of the infiltration line. Hachimi et al. [23] used cumulative infiltration data and empirical formula to simulate the soil infiltration line. Feng et al. [24] analyzed the microwave reflection waveform of layered soil by the theory of segmented transmission lines and predicted the position of the soil infiltration line by the waveform. Cui et al. [25] established a one-dimensional infiltration model based on the traveling characteristics of the wetting front (TCWF) model. The relationship between soil water infiltration rate and the soil infiltration line was analyzed with Hydrus-1D. Singh et al. [26] used the dimensional analysis to establish a model to simulate soil wetted depth and width. This novel model can determine the soil infiltration line more precisely. Although these studies have performed numerical simulation and experiments of soil infiltration line’s interaction [27], they ended without an accurate measurement method.

For the position measurement, Leuther et al. [8] analyzed the position of the soil infiltration line by X-ray under different initial water conditions in sewage irrigation. However, this expensive, radioactive, soil-devastating method is incompatible in practical application. Walker et al. [28] installed 5 Virrib soil moisture sensors at the horizontal depth of 10, 15, 20, 30, and 40 cm to monitor the position of the soil infiltration line. However, these sensors are difficult to install, can only measure the infiltration line at a specific point in the soil, and can significantly harm the soil structure. Monjezi et al. [29] conducted a drip irrigation experiment in a transparent box. He determined the actual position of the soil infiltration line by observing the infiltration line on the box wall and surface wetting area. However, due to the leakage in the wall of the test unit, this method cannot be applied effectively. Monceft et al. [30] determined the soil infiltration line by the visual method that requires excavation to form a soil profile. Topp et al. [31] proposed a method to measure the soil infiltration line by TDR (time domain reflectometry), with the influence of soil moisture on the amplitude of TDR waveform also studied. However, the long rise time of the excitation pulse signal in the Tektronix Model 7S12 caused obscure reflection of the resulting curve on the soil infiltration line’s position. Economically, the rise time of the step signal is not too fast in the mainstream TDR devices on the market. The step signal of the TDR device can be converted into a frequency domain signal through fast Fourier transformation (FFT). The amplitude and harmonic number of the frequency components will be attenuated proportionally. Low resolution and narrow bandwidth of TDR decrease the measurement accuracy. Therefore, when measuring soil infiltration lines, the waveform of TDR can only reflect the position of the starting point and ending point of the probe. Hence, this result cannot clearly reflect the waveform change caused by the soil infiltration line.

Based on the principle of impedance reflection and time-frequency conversion, a method for accurate measurement of the soil infiltration line using VFTT (the vector network analyzer’s frequency domain signals are converted to the time domain) technology is proposed in this paper. VFTT is an effective method where the portable VNA (vector network analyzer) is used as the signal transmitter, with a printed circuit board (PCB) parallel line probe used as the sensor. The amplitude of the sinusoidal excitation signal transmitted by the vector network analyzer (VNA) can remain relatively stable, with its frequency up to 20 GHz. It can generate enough high-frequency signals with high resolution and small influence by external factors and send them to the DUT (device under test). Thus, high-resolution TDR signal measurement can be realized through VFTT technology that can better display the position of the soil infiltration line in the waveform. Moreover, as the portable mobile VNA upgrades, this method has a good application space for high-resolution measurement of soil infiltration line position.

## 2. Materials and Methods

### 2.1. Conversion from Time Domain to Frequency Domain

During the measuring of the position of the soil infiltration line, the soil where the sensor probe is located is a non-uniform medium. To calculate the non-uniform transmission line, Lin [32] used a simple algorithm for the input impedance calculation. The algorithm starts from the end of the transmission line. Its process is as follows:

(1) $$Z_{in}\left(z_{n}\right)=Z_{L}$$

(2) $$Z_{in\;}\left(z_{i}\right)=Z_{c,i+1}\frac{Z_{in\;}\left(z_{i+1}\right)+Z_{c,i+1}\tanh\left(\gamma_{i+1}l_{i+1}\right)}{Z_{c,i+1}+Z_{in\;}\left(z_{i+1}\right)\tanh\left(\gamma_{i+1}l_{i+1}\right)}$$

(3) $$Z_{in\;}(0)=Z_{c,1}\frac{Z_{in\;}\left(z_{1}\right)+Z_{c,1}\tanh\left(\gamma_{1}l_{1}\right)}{Z_{c,1}+Z_{in\;}\left(z_{1}\right)\tanh\left(\gamma_{1}l_{1}\right)}$$

In the equation, $Z_{c,i}$ and $\gamma_{i}$ are the characteristic impedance and propagation constant of the section *i* respectively; $Z_{in}\;\left(Z_{i}\right)$ and $Z_{i}$ are the input impedance of the corresponding circuit at *i*. According to the boundary conditions at Z=0, the input impedance can be calculated as:

(4) $$V(0)=\frac{Z_{in}(0)}{Z_{s}+Z_{in}(0)}V_{s}$$

These data, measured by vector networks, are discrete frequency domain data. To obtain the corresponding time domain response, linear frequency modulation *Z* transform [33] (Chirp-Z transform) is used. The Chirp-z transform enables the signal conversion to be uniformly sampled on the *Z* plane. This spiral starts at any point and ends at another arbitrary point, as shown in Equation (5).

(5) $$\left\{ \begin{array}{l} X(z)=\sum_{n=0}^{n=N}x(n)\cdot z^{-n}\\ z=AW^{-k},\;k=0,1,\cdots M-1\\ A=A_{0}\cdot e^{j2\pi \theta_{0}},\;W=W_{0}\cdot e^{i2\pi \phi_{0}} \end{array}\right.$$

Among them, A is the starting point of the sampling; W is the interval between the sampling points. The frequency domain data is then transformed to the time domain by inverse Chirp-z transform. The inverse Chirp-z transformation is equivalent to the conjugate of the result of Chirp-z transformation of frequency domain data [34], as shown in Equation (6):

(6) $$\begin{array}{c} \sum_{n=0}^{N-1}x[n]z^{\left(-n\right)}=X[z]\\ \sum_{n=0}^{N-1}x^{*}[n]z^{\left(-n\right)}=X^{*}[z^{*}]\\ {\left(\sum_{n=0}^{N-1}x^{*}[n]z^{\left(-n\right)}\right)}^{*}=X^{*}[z^{*}]=\sum_{n=0}^{N-1}x[n]{\left[z^{*}\right]}^{\left[-n\right]} \end{array}$$

The Kaiser-Bessel window is performed on the obtained *S*_11_ parameters [35], and the window function is shown in Equation (7), where W(n) is the first kind of deformed zero-order Bessel function, and β is a freely selectable parameter.

(7) $$W(n)=\frac{I_{0}(\beta )\sqrt{1-{\left(1-\frac{2n}{N-1}\right)}^{2}})}{I_{0}(\beta )}$$

The amplitude of the pulse waveform in the time domain response obtained by the Chirp-z transformation will be less than 1. Therefore, for the frequency domain response with non-zero edge data, its time domain result needs to be corrected by a scale factor. Summing the window function can get the correct scale factor of the corresponding time domain transformation, and the results are shown in Equation (8):

(8) $$W_{0}=\frac{\Delta \omega }{2\pi }\sum_{n=-N}^{N}W(n\Delta \omega )$$

where W is the Kaiser window function, Δω is the discrete interval of frequency domain data, and $W_{0}$ is the defined scale factor. After renormalization, the time domain transform becomes Equation (9):

(9) $$f_{VNA}(t)=\frac{1}{W_{0}}\cdot \frac{\Delta \omega }{2\pi }\sum_{n=-N}^{N}F(n\Delta \omega )\cdot W(n\Delta \omega )\cdot e^{jn\Delta \omega t}$$

where F(nΔω) is discrete frequency domain data. By converting the time domain to frequency domain signal and impedance change, the position of signal change, the position of the soil infiltration line can be obtained. That is, we can accurately locate the position of the infiltration line in the actual measurement process.

### 2.2. Experimental Preparation

To study the practicability of the VFTT method in measuring infiltration lines of different soils, four different texture soil samples of Shaanxi Lou soil, Loess, Black soil, and Clay loam are selected. Their physical parameters are shown in Table 1.

**Figure 1:**
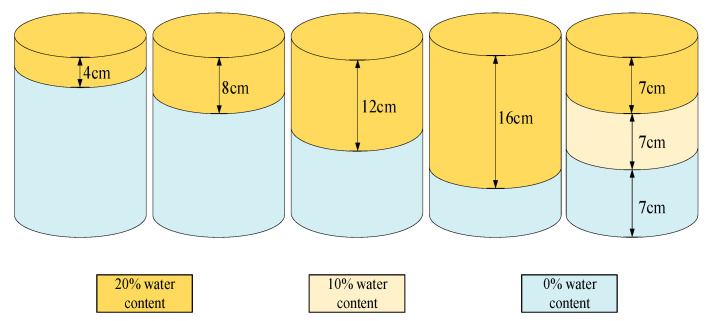
Schematic diagram of sample preparation.

The experiment was completed at an irrigation station (108°24′ E, 34°18′ N, altitude 521 m). The station is the Key Laboratory of Agricultural Water and Soil Engineering in Arid Areas of the Ministry of Education and belongs to Northwest A&F University. First, the soils were air-dried, ground, and refined. They were then sieved through a 1.0-mm (no. 18) sieve. Finally, the soil samples were oven-dried at 105 °C for 24 h and stored for experiment. Based on the density of each soil sample, deionized water was fully mixed to prepare soil samples with the volume water content of 0%, 10%, and 20%, respectively. To reduce the influence of ambient temperature, the experiment was carried out at an indoor temperature of 24 ± 2 °C. The soil samples were placed for more than 8 h before starting the measurement. They were then placed into polyvinyl chloride (PVC) pipes (210-mm height, 67.5-mm diameter). The two-layer soil columns’ filling heights of the upper and middle wet soil (20% volume water content) were controlled at 0, 4, 8, 12, 16, and 20 cm, respectively. The three-layer soil column’s upper, middle, and lower layers are 7 cm, with the soil of 20%, 10%, and 0% water content. Each layer is separated by PTFE (Poly tetra fluoroethylene), as shown in Figure 1.

**Figure 2:**
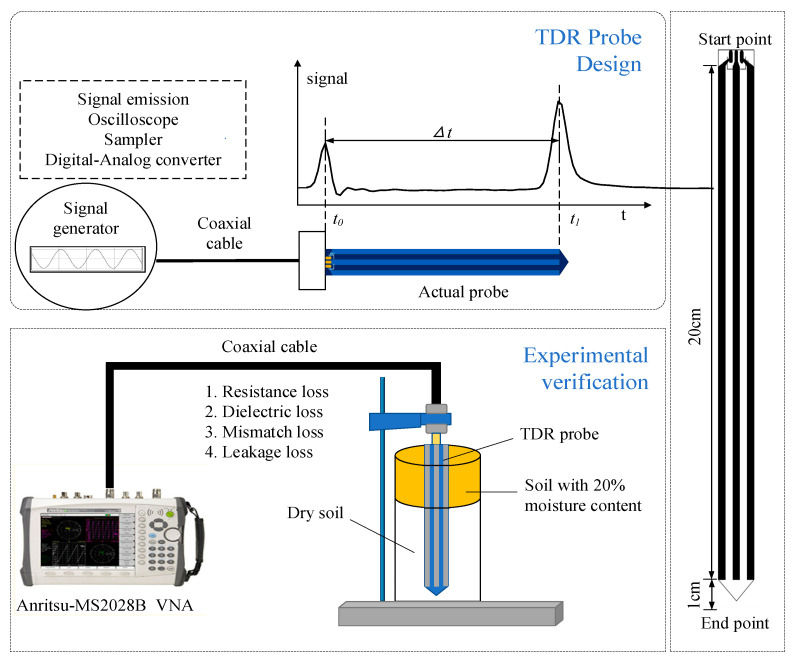
Schematic diagram of probe and measurement system.

In this experiment, the VNA is Anritsu-MS2028B, with the number of sampling points set to 1024, and the measurement frequency range 1 MHz~20 GHz. Before the test, the SOLT (Short-Open-Load-Thru) calibration method was adopted to calibrate the VNA [36]. The probe is a self-made three-pin probe made of PCB, with a length of 210 mm and a width of 20 mm. Three strip-shaped copper foils were laid on its surface, each with a length of 200 mm, a width of 4 mm, and a spacing of 4 mm. The schematic diagram of its design and measurement system is shown in Figure 2. Each soil sample was measured three times, with the average of the three values used as the final result. The actual water content was calculated by the oven drying method.

## 3. Results

The data processing and mapping were carried out by Origin 2018 and MATLAB 2018a. The linear correlation between the set value of soil infiltration line depth and the measured value obtained by the VFTT method was analyzed and evaluated, with determination coefficient (R^2^) and root mean square error (RMSE). The closer R^2^ is to 1 and RMSE is to 0, the more accurate the measurement method is.

### 3.1. Infiltration Line of Soil with Two-Layer

**Figure 3:**
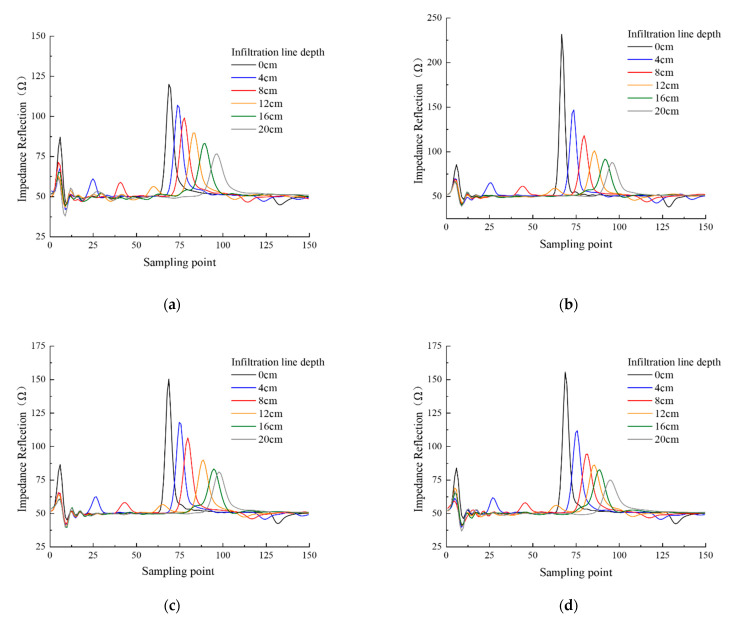
Measurement of infiltration lines with two layers for different soil: (**a**) Shaanxi Lou soil; (**b**) clay loam; (**c**) loess; (**d**) black soil.

The VFTT method is used to measure the permittivity of Shaanxi Lou soil, clay loam, loess soil, and black soil when the depth of infiltration line is 0, 4, 8, 12, 16, and 20 cm. The waveforms of each infiltration line depth are drawn, as shown in Figure 3.

**Figure 4:**
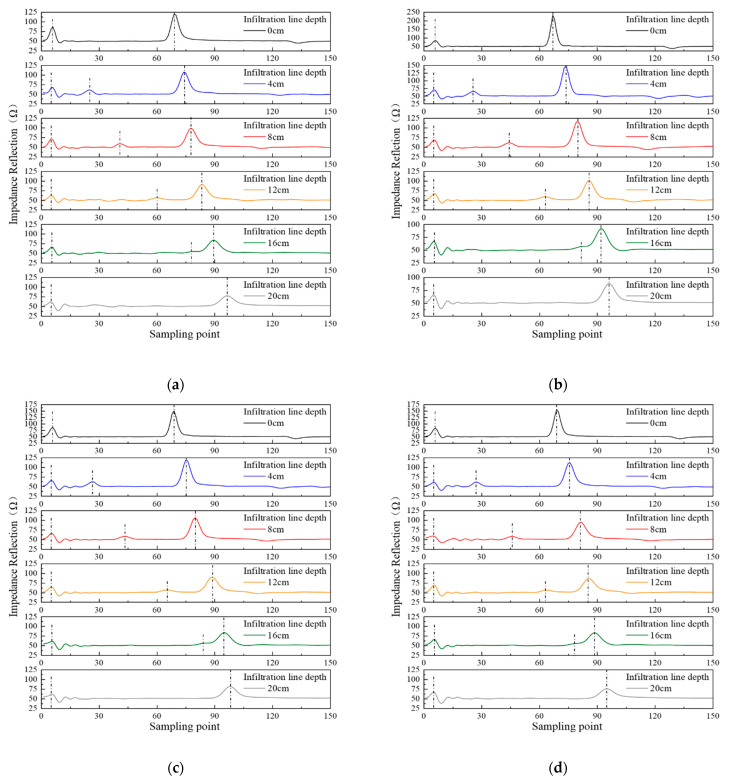
Single-wave measurements of different infiltration lines for four types of soil: (**a**) Shaanxi Lou soil; (**b**) clay loam; (**c**) loess; (**d**) black soil.

Figure 3 indicates the consistency of the four types of soils’ waves when measuring infiltration lines at different depths. The first reflection of microwave occurs when it passes through the interface between air and soil. This peak is the starting reflection point of TDR. When the microwave propagates to the soil infiltration line, it reflects and forms a reflection peak as the medium impedance changes, with the reflection point being the soil infiltration line’s position. Soil samples with different volumetric water content have different dielectric constants [37]. As a result, as the infiltration line deepens, the overall volume water content of the soil increases, with reduced signal propagation speed and backward-moving reflection peak at the probe’s end. To show the peak movement of soil impedance more clearly with the change of soil infiltration line depth, the measured waveforms of different soil infiltration line depths for each soil are shown in Figure 4.

Figure 4 reveals that the starting reflection peak of Shaanxi Lou soil, clay loam, loess, and black soil are all at the fifth sampling points. When there is no soil infiltration line in the soil samples, the medium where the probe is located is uniform, without reflection of the microwave signal. Therefore, the obtained TDR waveform only shows the two peaks at the starting and the end of the probe. When the soil infiltration line is 4, 8, 12, or 16 cm, the water content of the layered soil where the probe is located is different. This phenomenon caused the medium’s changes and the reflection of electromagnetic waves, so there is an additional infiltration line reflection peak between the starting reflection peak and the end one. The positions of each sampling point are shown in Table 2.

Table 2 displays the same initial reflection peak of the four soils, with slightly different end reflection points. That is caused by the non-uniform volumetric water content of the soil sample during the configuration process. By Equation (10), the distance between the initial reflection and the end reflection point can be taken as the probe length to normalize the sampling points. The measured values S of the infiltration lines’ positions at different depths can be obtained as following:

(10) $$s=\left(\frac{X_{i}-X_{start}}{X_{end}-X_{start}}\right)\cdot L\cdot 100\%$$

In this formula, L is the probe length; $X_{start}$ is the starting peak of reflection position; $X_{end}$ is the end reflection peak position; $X_{i}$ is the intermediate reflection peak position.

**Figure 5:**
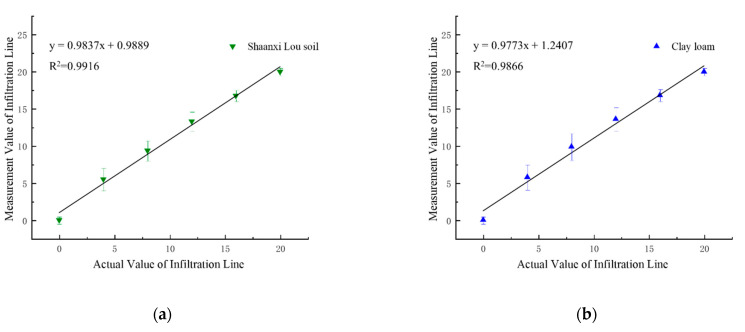

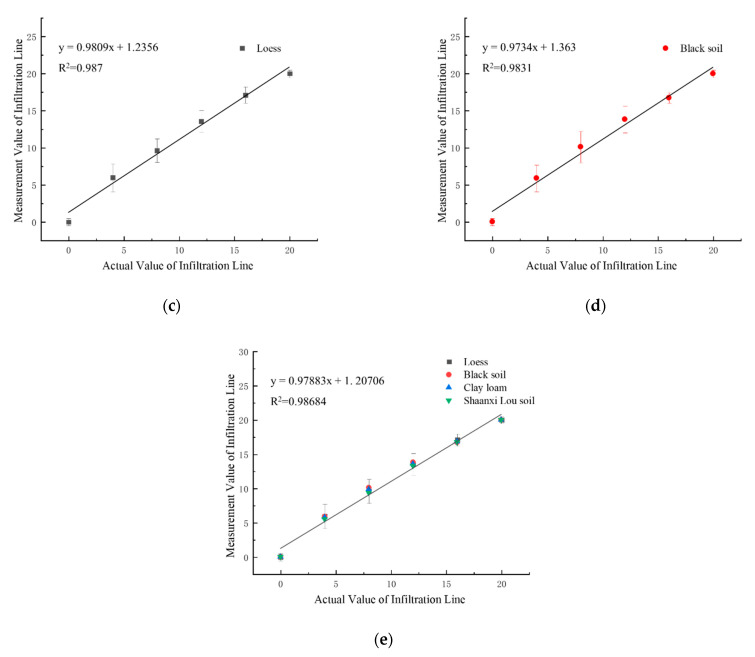
Correlation verification of two-layer soil infiltration line: (**a**) Shaanxi Lou soil; (**b**) clay loam; (**c**) loess; (**d**) black soil; (**e**) concatenated fitting of four soils.

The actual depth of the soil infiltration line is taken as the abscissa, with the measured value of the soil infiltration line as the ordinate. The soil infiltration lines’ positions for four different textures were fitted individually and concatenated, as shown in Figure 5.

The coefficient of determination (R^2^), model equation, and root mean square error (RMSE) are shown in Table 3.

Figure 5 and Table 3 show that the R^2^ of the actual and measured values for the soil infiltration line positions is above 0.98. With excellent correlation, this method can be used to find the position of the soil infiltration line accurately. Therefore, the infiltration line position of different soil can be accurately measured by the VFTT method.

### 3.2. Three-Layer Soil Infiltration Line Measurement

**Figure 6:**
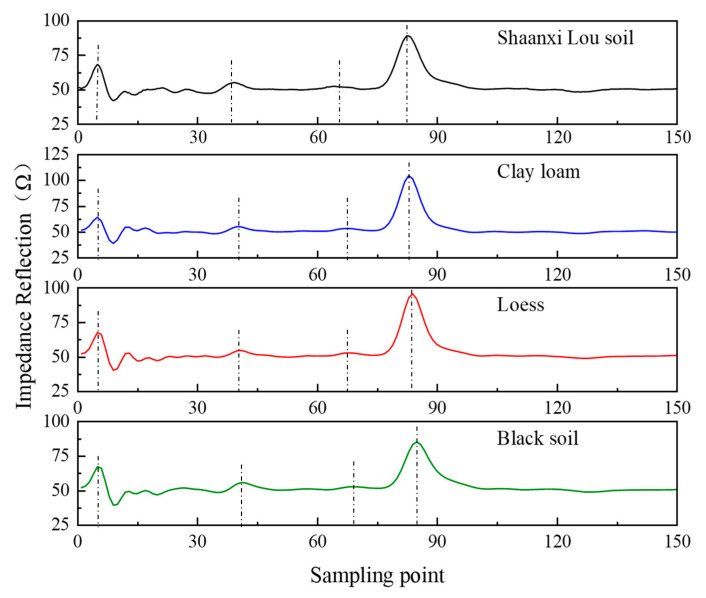
Three-layer soil infiltration line test.

Considering the gradual process of soil moisture as the soil moisture infiltrates and the applicable scope of soil infiltration in measurement, a three-layer soil infiltration experiment was set up in this study. The three-layer soil column’s upper, middle, and lower layers are all 7 cm, with soil containing volume water content of 20%, 10%, and 0%, respectively. The sampling points of the four kinds of soil are 1024, and the experiment results are shown in Figure 6.

Figure 6 shows two interfaces with discontinuous impedance in the three layers of soil. Therefore, there are two reflection points of the soil infiltration line between the initial and the end reflection points. In the two reflection positions of 7 cm and 14 cm, Shaanxi Lou soil is the 79th and 129th sampling points respectively, clay loam, the 40th and 67th sampling points respectively, loess, the 41st and 68th sampling points respectively, and black soil, the 41st and 69th sampling points respectively. At the same time, the total water content of the soil is the same because the water content of the upper, middle, and lower layers of the four types of soil are 20%, 10%, and 0%, respectively, with each layer’s height of the soil being 7 cm. However, the volume moisture content of soil samples is slightly different in the configuration process. It has a certain influence on the transmission speed of the electromagnetic wave, so the reflection point at the end of the probe is slightly different.

### 3.3. Universal Adaptability Analysis

**Figure 7:**
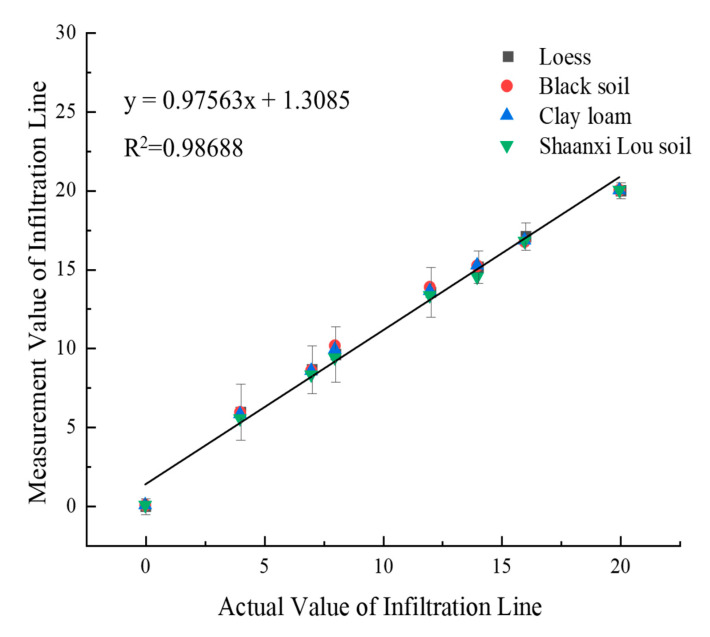
Correlation verification of multi-layer soil infiltration line.

To verify the adaptability and accuracy of the VFTT method in measuring the position of the soil infiltration line, this study verifies the correlation between the two-layer soil infiltration line experiment and the three-layer soil infiltration line test. The positions of the soil infiltration lines of four different textures are juxtaposed and fitted, with the results shown in Figure 7.

Figure 7 reveals that the test/actual values of the two-layer soil infiltration line and the three-layer soil one is fitted close to 1:1. The coefficient of determination can reach 0.985, with excellent correlation proven, indicating that the soil infiltration line measurement method transformed in the time domain and frequency domain is applicable in measuring water content infiltration line in different layers. Therefore, the position of the soil infiltration line can be accurately located by VFTT technology.

## 4. Conclusions

Aiming at the low resolution and narrow bandwidth of the existing time-domain reflectometer, this paper proposes a method to measure the soil infiltration line with VFTT accurately. Based on measuring soil water content by the dielectric method, the discrete frequency-domain data are converted to time-domain by combining with Chirp-Z transform. Compared with the general method, the VFTT method causes less damage to soil and can measure the position of the soil infiltration line more easily and accurately. This paper tested four kinds of soils—Shaanxi Lou soil, loess, clay loam, and black soil—for two and three layers of soil infiltration lines. The results show that the fitting degree between the actual and measured values at 0, 4, 7, 8, 12, 14, 16, and 20 cm is over 0.98, with excellent correlation. It means that VFTT can accurately determine the position of the soil infiltration line within 20 cm. This paper provides a simple method for soil moisture monitoring in water-saving irrigation and can help improve the automation and intelligence of agricultural irrigation system, which is significant for the development of water-saving agriculture.

## Figures and Tables

**Table 1:** Physical properties of four different texture soils.

Soil Type	Cosmid (%) (<0.002 mm)	Powder (%) (0.002~0.02 mm)	Sand Grain (%) (0.02~2 mm)	Bulk Density (g/cm^3^)
Shaanxi Lou soil	35.23	46.12	18.65	1.26
Clay loam	23.02	27.38	50.60	1.37
Loess	19.44	22.32	58.24	1.50
Black soil	14.45	31.12	54.43	1.36

**Table 2:** The position of the starting point, intermediate reflection point, and the end point of the four soils with different depth of infiltration lines.

	Depth		0 cm	4 cm	8 cm	12 cm	16 cm	20 cm
Soil Type								
Shaanxi Lou soil		Starting point	5	5	5	5	5	5
		Intermediate reflection point		25	41	60	80	
		End point	67	74	78	83	90	97
Clay loam		Starting point	5	5	5	5	5	5
		Intermediate reflection point		26	44	63	82	
		End point	69	74	80	86	92	96
Loess		Starting point	5	5	5	5	5	5
		Intermediate reflection point		26	43	65	82	
		End point	69	75	80	89	95	98
Black soil		Starting point	5	5	5	5	5	5
		Intermediate reflection point		27	46	64	79	
		End point	69	76	82	86	89	95

**Table 3:** Correlation table of four kinds of soil infiltration lines.

Soil Type	Equations of Model	R^2^	*RMSE*
Shaanxi Lou soil	y = 0.9856x + 0.9668	0.991	0.678
Clay loam	y = 0.9795x + 1.2151	0.9855	0.798
Loess	y = 0.9835x + 1.2068	0.986	0.653
Black soil	y = 0.9756x + 1.3365	0.9818	0.410
Concatenated fitting	y = 0.98x + 1.3345	0.9861	0.621

## Data Availability

The data presented in this study are available on request from the corresponding author.
